# Case Report: Multiple radiotherapy courses for extensive recurrent cutaneous metastases from colorectal cancer

**DOI:** 10.3389/fonc.2026.1910929

**Published:** 2026-10-02

**Authors:** Mehdi Belbachir, Nicolas Danthez, Cécile Torregrosa, Gian Guyer, Rémy Kinj

**Affiliations:** 1 Department of Radiation-Oncology, Lausanne University Hospital (CHUV), Lausanne, Switzerland; 2 Institute of Radiation Physics, Lausanne University Hospital (CHUV), Lausanne, Switzerland; 3 Faculty of Biology and Medicine, University of Lausanne, Lausanne, Switzerland

**Keywords:** cutaneous metastasis/metastases, palliative radiotherapy, rectal cancer, skin metastasis/metastases, multiple radiotherapies, tomotherapy

## Abstract

We report the management of a 36-year-old man presenting with rapidly progressive and highly symptomatic cutaneous metastases (CMs) from metastatic colorectal cancer (mCRC). He was referred to palliative radiotherapy because of painful and pruritic skin lesions. His history included two synchronous colorectal adenocarcinomas. Comprehensive next-generation sequencing identified the rectosigmoid tumor as the metastatic source and revealed a pathogenic germline BRCA1 mutation, supporting a precision systemic strategy (olaparib plus pembrolizumab). CMs were the patient’s dominant source of morbidity and progressively involved new skin sites; the patient therefore, received three sequential courses of helical tomotherapy (Radixact^®^), each delivering 30 Gy in 10 fractions to extended superficial volumes exceeding 50 cm. Each course treated adjacent fields with limited high-dose overlap. Deformable co-registration with the prior plans and cumulative-dose review confirmed that the high-dose overlap was limited, distinguishing this approach from re-irradiation. A posterior skin strip was consistently spared to preserve lymphatic drainage and reduce the risk of compartment toxicity. All three courses produced a very good partial response (VGPR) at six weeks, with toxicity limited to grade 1 dermatitis (no grade ≥2). Crucially, repeated irradiation maintained durable symptom control, avoided escalation of opioid analgesia for approximately six months, and allowed continuation of the same systemic regimen for nine months until lung progression. This case illustrates that iterative skin-directed radiotherapy can deliver safe, repeatable, high-quality palliation in extensive CMs.

## 1 Introduction

### 1.1 Cutaneous metastases: clinical overview and therapeutic challenges

Cutaneous metastases (CMs) are a rare oncological entity that can occur in nearly all types of cancer (1, 2). The reported prevalence ranges from 0.7% to 9%, most frequently in the context of widespread metastatic disease (1, 3). CMs may display a wide range of dermatological appearances (2). In colorectal cancer, these lesions typically present as firm, painless subcutaneous or intradermal nodules measuring a few centimeters that tend to coalesce (4). When associated with lymphatic involvement, CMs can lead to pain, pruritus, and significant functional discomfort. Treatment primarily relies on systemic therapies, which are often of limited efficacy in CMs, whereas skin-directed approaches may offer meaningful local control and symptom relief (5).

While radiotherapy is well established for palliation of bone and brain metastases (6–9), prospective data supporting its use in extensive CMs remain scarce (5). Nevertheless, radiotherapy has the unique ability to treat lesions at varying depths by tailoring the x-ray beam modality and energy, making it a versatile and effective option for extensive cutaneous disease (10).

### 1.2 Rationale for the case report

We describe the unusual case of a young patient who developed rapidly progressive and extensive CMs following surgical resection of two synchronous primary colorectal cancers (CRC). The extensive skin involvement, which developed over a short period, made CMs the patient’s primary source of morbidity, and these responded mainly to radiotherapy.

## 2 Case presentation

A 36-year-old man was referred to our institution. His history included a melanoma *in situ*, diagnosed three years earlier and treated with local excision without recurrence. He worked as an information technology specialist, lived with his partner, and had one child. There was no family history of cancer.

Approximately 4 years before presentation, the patient underwent upfront surgical management with total proctocolectomy for two synchronous colorectal adenocarcinomas. The first tumor, located at the rectosigmoid junction, was staged pT4a pN2 (27/35) cM0, grade 3, mismatch repair (MMR)-proficient, KRAS, NRAS, and BRAF wild type, without vascular or perineural invasion. The second tumor, arising in the ascending colon, was staged pT4a pN0 (0/30) cM0, grade 2, MMR-proficient, and harbored a BRAF V600E mutation.

Despite curative-intent surgery, the patient rapidly developed metastatic colorectal cancer (mCRC), predominantly in supraclavicular lymph nodes (**Figures 1A1, A2**). The spread was associated with the progression of the KRAS wild-type rectosigmoid cancer, as confirmed by lymph node biopsy. He subsequently received multiple lines of systemic therapy, including folinic acid, fluorouracil, and irinotecan (FOLFIRI) plus bevacizumab, then cetuximab, and finally TAS-102 combined with bevacizumab.

**Figure 1:**
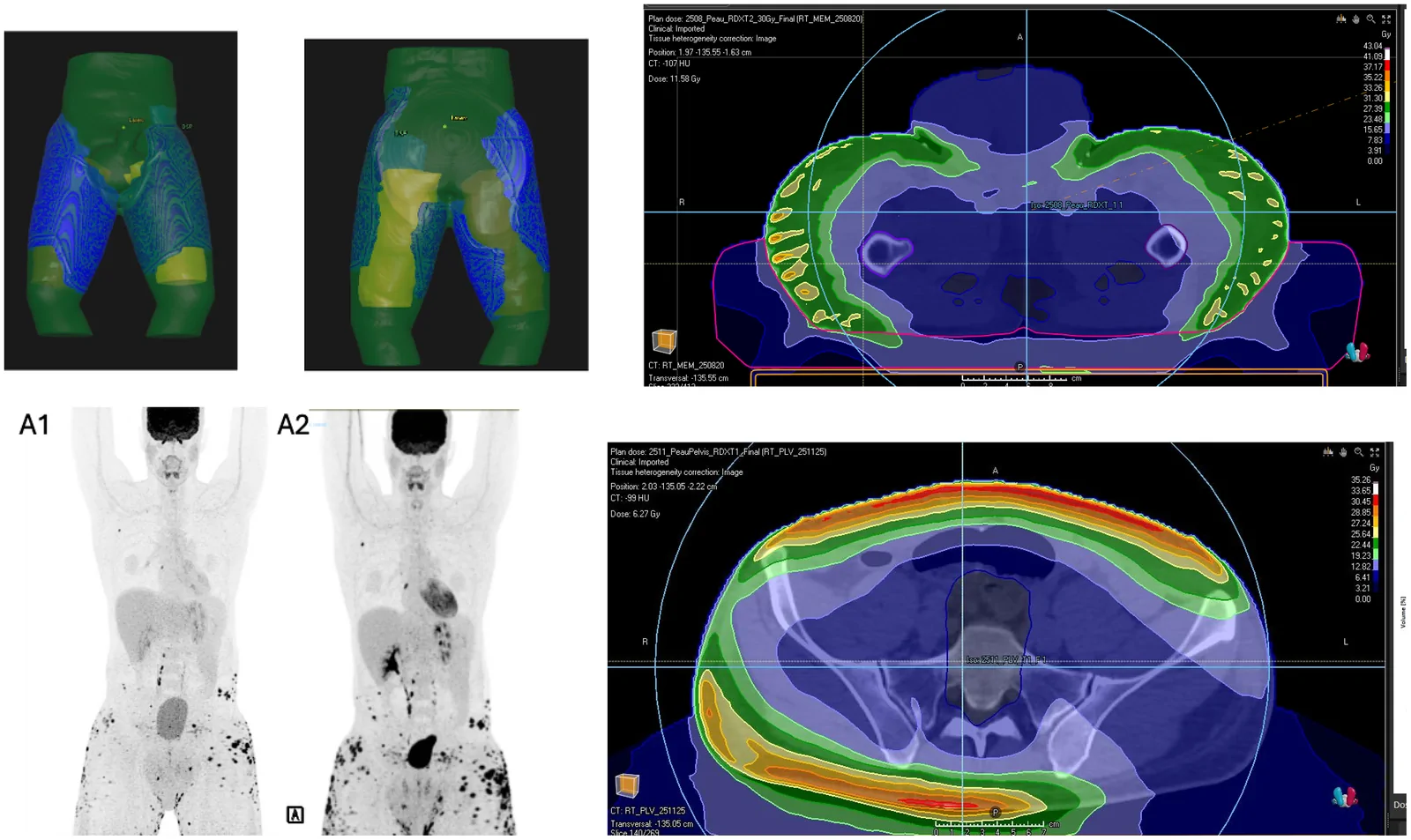
First-course imaging and treatment planning. **(A1, A2)** Serial ¹^8^F-fluorodeoxyglucose (¹^8^F-FDG) PET/CT maximum-intensity projections [**(A1)** 16.07.2025; **(A2)** 29.08.2025] showing rapid progression of cutaneous and nodal disease over six weeks. **(B)** CTV/PTV delineation on both thighs by helical tomotherapy (Radixact^®^); the posterior thigh strip (lesion-free) was deliberately spared to preserve lymphatic drainage and limit the risk of compartment toxicity. **(C, D)** Axial dose distributions across the thighs **(C)** and pelvis **(D)** for the first course (30 Gy/10 fractions prescribed to the PTV D50%), showing the superficial high-dose region and rapid fall-off with depth.

A few years after metastatic spread, while on TAS-102 plus bevacizumab, he developed rapidly progressive, painful, and pruritic CMs. These involved the lower back, the pelvis, and both thighs, which had pre-existing lymphedema. Clinically, the lesions appeared as non-specific, slightly indurated papules and nodules without epidermal involvement (**Figure 2A**). Given their atypical appearance and the patient’s history of melanoma *in situ*, histological confirmation was required. Skin biopsy with immunohistochemistry confirmed a colorectal origin. The full timeline is shown in **Figure 3**.

**Figure 2:**
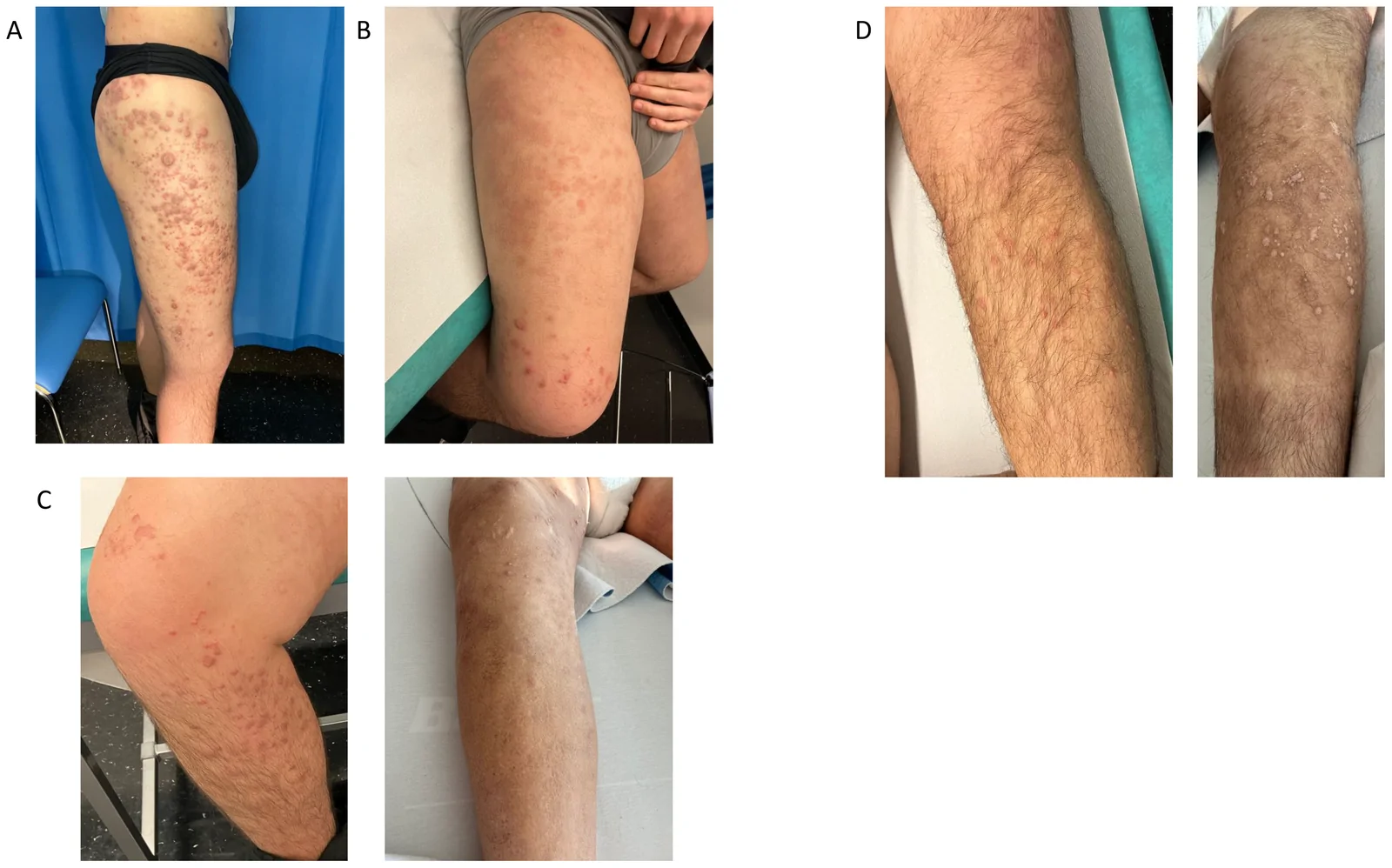
Multiple photographs illustrating the outcomes: **(A)** Medio-anterior and lateral views of the right thigh, right at the end of the first course (30 Gy/10 fractions). Confluent erythematous papules and nodules cover the anterolateral thigh and extend into the inguinal fold, on a background of diffuse erythema corresponding to grade 1 dermatitis. **(B)** The same region six weeks later, with a bilateral and side view of the right thigh. The nodules have flattened almost completely, leaving faint erythematous macules and a mild post-inflammatory hyperpigmentation. No residual palpable nodularity was present, and the treated skin remained intact throughout, without ulceration or desquamation. **(C)** Second course, right leg: baseline (left) and five months after treatment (right). At baseline, discrete indurated nodules are visible over the anterior and lateral aspect of the leg. At five months, the nodules have resolved; the treated field shows diffuse hyperpigmentation and alopecia, both grade 1 late radiation effects. **(D)** Third course, left leg: baseline (left) and two months after treatment (right). The multiple nodules seen at baseline have regressed, again leaving a well-demarcated area of hyperpigmentation, alopecia, and dry skin corresponding to the treated volume.

**Figure 3:**
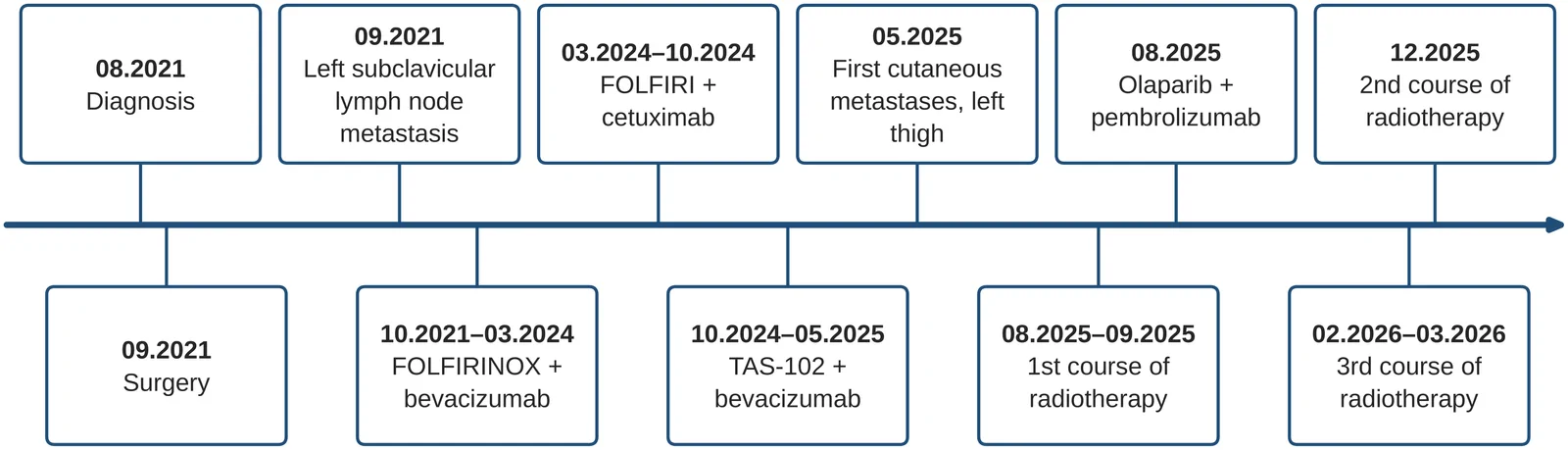
Patient timeline from diagnosis and surgery (2021) through successive systemic lines to the three radiotherapy courses (2025–2026).

To determine the primary source of dissemination, comprehensive next-generation sequencing (NGS) was performed, including a 400-gene panel and homologous recombination deficiency (HRD) analysis. The CMs harbored a TP53 p.Arg249Gly mutation and showed no BRAF mutation, matching the molecular profile of the rectosigmoid primary. Furthermore, germline testing confirmed that the patient was a heterozygous carrier of the pathogenic BRCA1 variant (c.3286_3289del, p.Gln1096Valfs*12). Tumor HRD analysis (27-gene homologous recombination repair panel) detected the same BRCA1 variant at a markedly elevated allelic frequency (76%, for an estimated tumor cellularity of approximately 70%), consistent with loss of the wild-type allele in the tumor. This was accompanied by a high genome-wide Large Scale Transitions (LST) score (LST = 37). Together, these findings provided converging genomic evidence of biallelic BRCA1 inactivation and of homologous recombination deficiency at the tumor level, beyond the germline variant alone.

These findings prompted a multidisciplinary tumor board discussion, at the conclusion of which olaparib combined with pembrolizumab was initiated in place of standard fruquintinib (11), on the basis of emerging data from the KEYLYNK-007 trial (12).

The patient was referred for palliative radiotherapy because of severe pain and pruritus in both thighs. CT simulation was performed using a Vac-Lok immobilization system to ensure reproducible positioning. Radiopaque wires were placed on the skin to delineate clinically involved areas. No bolus was applied, given the extent of the area requiring irradiation. The clinical target volume (CTV) encompassed the clinically visible cutaneous lesions and was expanded by a 7 mm margin to a planning target volume (PTV), reaching approximately 12 mm below the skin surface. This margin aimed to cover potential dermal and subcutaneous tumor infiltration while preserving deeper tissues, in line with the palliative intent. A thin strip of posterior thigh skin was deliberately spared from high-dose irradiation to preserve lymphatic drainage and reduce the risk of compartment syndrome. Irradiating the full circumference of a limb carries a risk of circumferential subcutaneous fibrosis, which can impair lymphatic and venous return and aggravate pre-existing lymphedema, producing a chronic compartment syndrome with pain, swelling, and functional impairment. Sparing a longitudinal strip of skin preserves a drainage corridor and is standard practice in limb sarcoma radiotherapy (13).

Helical tomotherapy was selected for its ability to treat long, continuous target volumes of up to 50 cm without field junctions (**Figure 1B**). A total dose of 30 grayGy in 10 fractions was prescribed (**Figure 1C**). This fractionation is our institutional standard for palliative radiotherapy; it offers a good balance between tumor control and late toxicity. A higher dose per fraction would have carried a greater risk of cutaneous fibrosis, which could have worsened the pre-existing lower-limb lymphedema and further impaired the patient’s quality of life.

The dose was prescribed to the median dose (D50%) of the PTV, ensuring appropriate coverage of superficial lesions while avoiding excessive hotspots. The dose distribution showed a marked gradient, with high-dose regions confined to superficial tissues and a rapid fall-off within the first centimeters of depth, sparing deeper tissues. To prevent compartment syndrome, we took care not to exceed a circumferential dose of 42.75 Gy (13). Treatment was delivered using an intermittent split-course schedule (four consecutive treatment days followed by a four-day break) to allow skin recovery, particularly in the genital region (**Figure 1D**). Treatment sessions lasted 32 minutes, which required careful attention to the patient’s comfort and immobilization. Acute toxicity, graded according to the Common Terminology Criteria for Adverse Events (CTCAE) v6.0, comprised grade 1 radiation dermatitis of the thighs and the inguinal folds and grade 1 alopecia. A transient resurgence of pruritus was noted during follow-up; it resolved with antihistamines. No higher-grade toxicities, including compartment syndrome, were documented. Standard topical skin care and close nursing follow-up ensured satisfactory symptom control throughout. Six weeks after treatment, the nodules had shrunk significantly and some had disappeared (**Figure 2B**); this was recorded as a very good partial response (VGPR) at follow-up, which consisted of clinical examination by the radiation oncologist. VGPR was defined as a significant reduction in the size of the CMs on physical examination. No Response Evaluation Criteria in Solid Tumors (RECIST) assessment was applicable, as the lesions were superficial, multiple, and partly confluent, and were not measurable on cross-sectional imaging. We therefore acknowledge that this is a subjective clinical assessment rather than a validated response criterion.

Pain decreased to grade 0, with the patient using only short-acting morphine drops. Pruritus in the treated area also decreased to grade 0.

Three months after the initial course, the patient again developed pruritus and pain associated with growing nodules on the right leg, lower abdomen, and lower back (**Figure 2C**). He was referred to us for a second course of radiotherapy. Two simulation scans were acquired. The first was a pelvic acquisition, performed head-first with leg and foot cushions, covering the lower back and lower abdomen; the second targeted the right leg and was performed feet-first, with the treated limb immobilized in a vacuum-fixation device. During the leg acquisition, the VGPR obtained after the first course made it straightforward to distinguish the new growing nodules from previously irradiated lesions. No bolus was used. Given the good tolerance of the first course, the schedule was shortened to five days per week, with 10 fractions of 3 Gy each. The CTV was defined from the surface radiopaque wires as a 5-mm-deep volume; a posterior skin strip was again spared, in continuity with the right thigh corridor from the first course. A 5-mm inward margin was added to generate the PTV. For planning, deformable image registration (DIR) with the CT of the first plan was performed using the ANACONDA algorithm implemented in RayStation (14), with the default deformation strategy and a 0.25-cm-resolution grid. The previously treated volumes were transferred to the new CT using the DIR, which showed that the new volumes overlapped with the first ones on the left flank and the right knee. Physical dose summation was performed with the new dose (prescribed to the D50%). In addition, an equivalent dose in 2 Gy fractions (EQD2) dose calculation was performed using an alpha/beta value of 3 Gy for all tissues. The maximal cumulative doses in the overlapping fields were a V60 at 235 cc and a Dmax at 77 Gy in EQD2. The second course was well tolerated. The patient reported grade 1 dermatitis during treatment and grade 1 dry skin and alopecia six weeks later, all managed with local care. The response was similar to that of the first course, with a significant reduction in nodule size, again recorded as a VGPR. Pain in the gluteal region decreased. The morphine dose remained unchanged for several months thereafter (**Table 1**).

**Table 1:** Iterative palliative radiotherapy courses: treatment, response, and tolerance.

	Course 1	Course 2	Course 3
Timing	Baseline	3 months after Course 1	6 months after Course 1
Site(s) treated	Lateral pelvis + both thighs	Lower back + Middle Pelvis + right leg	Left lower leg
Dose / fractionation	30 Gy / 10 fr/ 4 d a w	30 Gy / 10 fr/ 5 d a w	30 Gy / 10 fr/ 5 d a w
Technique	Helical tomotherapy (Radixact); posterior skin-strip sparing	Helical tomotherapy; two isocenters (pelvis head-first; right leg feet-first), skin strip sparing	Helical tomotherapy; posterior skin-strip sparing
Response at 6 weeks	Very good partial response	Very good partial response	Very good partial response
Response at 2 months	Very good partial response	Very good partial response	Very good partial response
Cinical outcomes	Resolution of pain and pruritus to grade 0	Pain Grade 1 and pruritus grade 0	Pain grade 1 and pruritus grade 0
Acute toxicity	Grade 1 dermatitis (inguinal folds); no Grade 2	Grade 1 dermatitis; no Grade 2	Grade 1 dermatitis; no Grade 2
Late toxicity (~3 mo)	Grade 1 hyperpigmentation (lower limbs); dry skin Grade 1, alopecia Grade 1	Grade1 hyperpigmentation (right leg); Grade 1 dry skin, alopecia Grade 1	Grade1 hyperpigmentation; Grade 1 dry skin, alopecia Grade 1
Opioid analgesia	Oral morphine solution (drops), low dose	Sustained-release morphine 20 mg AM / 30 mg PM (50 mg/day)	Sustained-release morphine 20 mg AM / 30 mg PM (50 mg/day)
Cumulative-dose check (EQD2, α/β = 3)	Not applicable	Right knee and left abdominal flank V60Gy : 235 cc Dmax: 77 gy	Minimal overlap with left thigh; Dmax in new target 50 Gy
Systemic therapy	Olaparib + pembrolizumab	Continued	Continued

G, grade Gy, gray; fr, fractions; AM, morning; PM, evening; G1/G2, CTCAE grade 1/2. D a w, days a week schedule; Dmax, maximal dose at one point; V60, the volume receiving 60 grays. Consolidates the three iterative courses, summarizing their timing, treated sites, dose and fractionation, technique, response at six weeks and two months, acute and late toxicity, opioid requirement, cumulative-dose checks and concurrent systemic therapy, and confirms repeated very good partial responses with toxicity consistently limited to grade 1. A very good partial response means that the CMs shrank significantly on physical examination.

Three months after the second course (six months after the first), the patient developed new painful, pruritic, growing nodules on the left leg (**Figure 2D**) and was referred for a third course; olaparib had been maintained throughout. Simulation was performed feet-first in a vacuum-fixation device, with radiopaque markers placed around the symptomatic area. The durable response to the first course facilitated the distinction between progressing and stable nodules. No bolus was used. Deformable fusion with the CTs of the first and second plans was performed using the same strategy as described above. There was limited overlap with the previously treated left thigh region, which we monitored carefully. The CTV encompassed the full circumference of the leg as a 5-mm-deep volume, with a small posterior skin strip spared in continuity with that of the left thigh from the first course; this corridor was free of CMs. A 5-mm depth margin was added to create the PTV. The dose was prescribed to the D50%: 30 Gy in 10 daily fractions, five fractions per week. Dose summation using the DIR of the prior doses was performed for the physical dose and for the EQD2 dose using an alpha/beta value of 3 Gy. The maximum dose in the new target was 50 Gy in EQD2 at the left knee. The third course was well tolerated; the patient experienced grade 1 dermatitis at the end of the treatment and, 6 weeks later, grade 1 hyperpigmentation, grade 1 alopecia, and grade 1 skin dryness. The nodules almost vanished in the lower leg, which was recorded as a VGPR at the 2-month follow-up; pruritus was graded 0 and pain decreased.

Outcomes and toxicities are summarized in **Table 1**. Acute tolerance was favorable after each course. The patient consistently reported grade 1 radiation-induced side effects, most pronounced in the inguinal folds, which resolved with local care (emollient cream and antiseptics); no grade 2 or higher dermatitis occurred at any point. Late toxicity was likewise limited. The symptomatic benefit obtained allowed us to avoid any escalation of opioid analgesia for approximately six months. This was clinically meaningful for the patient, who had previously experienced opioid-induced constipation, and it helped preserve his quality of life even as his disease progressed. Oncologic progression in this patient was driven predominantly by the CMs, which were successfully controlled by radiotherapy; iterative irradiation therefore allowed us to maintain the same systemic regimen for nine months despite disease progression. Eight months after the first course, the disease spread to the lungs, prompting a switch to fruquintinib.

## 3 Discussion

CMs are generally associated with advanced disease and poor prognosis, with reported median survival ranging from 3 to 18 months (15, 16). The pathophysiology involves several potential pathways, including direct extension, lymphatic spread, and hematogenous dissemination (4). In this case, the extensive involvement of the lower half of the body and the presence of underlying lymphedema strongly suggested massive lymphatic infiltration.

The palliative management of cutaneous metastases aims primarily to alleviate pain, pruritus, and exudation in order to improve quality of life (17, 18). A meta-analysis by Spratt et al. showed that radiotherapy can be highly effective for skin lesions, yielding an objective response rate of up to 83.8% (5). Similarly, Reinking et al. reported that predefined therapeutic goals (pain reduction, tumor mass regression, or cessation of bleeding) were achieved in 85% of patients treated with radiotherapy for malignant wounds (17).

To the best of our knowledge, and based on the published cases identified in our review, this is the first report of multiple iterative skin irradiations for symptomatic CMs in mCRC (**Supplementary Table 1**). We acknowledge that most published cases focus mainly on the diagnostic aspects of the disease and provide little detail on subsequent treatment.

In mCRC, precise localization of the primary tumor and comprehensive molecular characterization are crucial, as distinct tumor clones may exhibit divergent biological behavior and therapeutic sensitivity. In this case, NGS analysis of the cutaneous metastasis proved to be decisive, demonstrating the absence of a BRAF mutation and the presence of a TP53 p.Arg249Gly mutation. This molecular profile matched that of the rectosigmoid primary tumor, thereby formally identifying it as the source of metastatic spread.

Furthermore, subtraction analysis of the NGS data enabled identification of a pathogenic germline BRCA1 mutation (c.3286_3289del), thereby allowing the clinical team to move beyond standard third-line therapeutic options for mCRC such as fruquintinib. We acknowledge, however, that this constitutes an off-label use, and that decisions of this nature warrant careful discussion in dedicated multidisciplinary meetings.

CMs can be a leading cause of poor quality of life in cancer patients (19). This case illustrates that extensive symptomatic cutaneous metastases can be effectively palliated using advanced radiotherapy techniques, even when very large treatment volumes are required. When combined with comprehensive molecular profiling and multidisciplinary decision-making, such approaches can substantially improve quality of life in patients with mCRC whose life expectancy has improved markedly over the past decade (20).

## 4 Patient perspective

The patient expressed satisfaction with the procedure and outcome and was thankful for the treatment, radiotherapy being the only treatment to alleviate all his symptoms at once (itchiness, pain, and exudation).

## Data Availability

The original contributions presented in the study are included in the article/**Supplementary Material**. Further inquiries can be directed to the corresponding author.

## Supplementary material

Supplementary Table 1 Reported cases of cutaneous metastases from colorectal cancer, with skin-directed radiotherapy exposure. Compiled from the case series and reviews of Dehal et al. (2016) (16), Hakami et al. (2020) (21), and Ahmed et al. (2020) (4), with the present case added. Cases are ordered chronologically; the present case appears last.
