# Supplementary material for: Case Report: Multiple radiotherapy courses for extensive recurrent cutaneous metastases from colorectal cancer
Source: Front Oncol. 2026 Oct 2;16:1910929. doi: 10.3389/fonc.2026.1910929 (PMC13672187; doi:10.3389/fonc.2026.1910929)
Supplement: Supplementary file 1 [file Table1.docx]

| **Author, year** | **Age** | **Sex** | **Histology** | **Skin metastasis location** | **Morphology** | **Skin-directed treatment** | **RT**  **(number of courses)** | **Survival/follow up**  **months** | **Source review** |
| --- | --- | --- | --- | --- | --- | --- | --- | --- | --- |
| Gray & Das, 1989 | 79 | F | Adenocarcinoma | Leg | Nodules | None | None | 18 months | Dehal 2016 |
| De Friend et al., 1992 | 49 | F | Adenocarcinoma | Perineum | Nodules | Surgery | None | NR | Dehal 2016 |
| Reed & Stoddard, 1992 | 68 | F | Adenocarcinoma, poorly diff. | Perineum | Nodules | Surgery | None | NR | Dehal 2016 |
| Kauffman & Sina, 1997 | 50 | M | Adenocarcinoma, signet ring | Multiple | Plaques | None | None | 3 months | Dehal 2016 |
| Adani et al., 2001 | 70 | F | Adenocarcinoma | Leg | Nodules | Chemoradiation | Single course | 14 months | Dehal 2016 |
| Melis et al., 2002 | 41 | M | Adenocarcinoma | Perineum | Plaques | None | None | NR | Dehal 2016 |
| Tsai et al., 2002 | 47 | M | Adenocarcinoma, signet ring | Multiple | Nodules | Chemotherapy | None | 4 months | Dehal 2016 |
| Damin et al., 2003 | 44 | M | Adenocarcinoma | Groin | Zosteriform | Radiotherapy | Single course | 5 months | Dehal 2016 |
| Hayashi et al., 2003 | 50 | M | Adenocarcinoma, mucinous | Perineum | Nodules | None | None | NR | Dehal 2016 |
| Sarid et al., 2004 | 60 | F | Adenocarcinoma, mucinous | Chest, abdomen | Ulcers | Surgery | None | 56 months | Dehal 2016 |
| Kilickap et al., 2006 | 29 | M | Adenocarcinoma, signet ring | Chest wall, axilla | Nodules | Surgery+ Chemotherapy | None | 4 months | Dehal 2016 |
| Reuter et al., 2006 | 69 | M | Adenocarcinoma | Perineum | Plaques | None | None | 6 months | Dehal 2016 |
| Tan et al., 2006 | 70 | M | Adenocarcinoma, mucinous | Back | Nodules | Surgery then chemo | None | NR | Dehal 2016 |
| Tan et al., 2006 | 53 | F | Adenocarcinoma | Perineum | Nodules | Surgery then chemoradiation | Single course | 26 months | Dehal 2016 |
| Nasti et al., 2007 | 76 | F | Adenocarcinoma | Parotid skin + forehead | Nodules | None | None | 15 months | Hakami 2020 |
| Gazoni et al., 2008 | 55 | F | Adenocarcinoma, poorly diff. | Perineum | NR | Chemoradiation | Single course | 3 months | Dehal 2016 |
| Gazoni et al., 2008 | 66 | M | Adenocarcinoma, poorly diff. | Perineum | NR | Chemoradiation | Single course | 4 months | Dehal 2016 |
| Gazoni et al., 2008 | 68 | M | Adenocarcinoma, poorly diff. | Thigh, axilla | NR | Chemoradiation | Single course | 3 months | Dehal 2016 |
| Gazoni et al., 2008 | 72 | M | Adenocarcinoma | Perineum | NR | Chemoradiation | Single course | 5 months | Dehal 2016 |
| Gazoni et al., 2008 | 65 | M | Adenocarcinoma | Perineum | NR | Chemoradiation | Single course | 7 months | Dehal 2016 |
| Gazoni et al., 2008 | 78 | M | Adenocarcinoma | Perineum | NR | Chemoradiation | Single course | 1 month | Dehal 2016 |
| Tranchart et al., 2008 | 59 | F | Adenocarcinoma, well diff. | Perianal | Nodules | Surgery and chemotherapy | None | 16 months | Hakami 2020 |
| Tranchart et al., 2008 | 70 | M | Adenocarcinoma, well diff. | Perianal | Nodules | Surgery | None | 22 months | Hakami 2020 |
| McWeeney et al., 2009 | 72 | M | Adenocarcinoma | Perineum | Nodules | Surgery | None | NR | Dehal 2016 |
| Saladzinskas et al., 2010 | 64 | M | Adenocarcinoma, mucinous | Face | Ulcers | Surgery | None | 7 months | Dehal 2016 |
| Goris et al., 2011 | 79 | M | Adenocarcinoma | Pubis, penis, scrotum | Nodule | None | None | 6 months | Hakami 2020 |
| Horiuchi et al., 2011 | NR | NR | Adenocarcinoma | Scalp | NR | None | None | Poor (months NR) | Ahmed 2020 |
| Ismaili et al., 2011 | 50 | F | Adenocarcinoma, signet ring | Multiple | Zosteriform | None | None | 1 month | Dehal 2016 |
| Balta I et al., 2012 | 46 | M | Adenocarcinoma, mucinous | Perineum | Ulcers | None | None | NR | Dehal 2016 |
| Nguyen et al., 2012 | 65 | M | Adenocarcinoma | Right flank | NR | Chemotherapy | None | Poor (months NR) | Ahmed 2020 |
| Relles et al., 2012 | 55 | M | Adenocarcinoma | Right upper lip | NR | Surgery | None | Poor (months NR) | Ahmed 2020 |
| Akpak et al., 2013 | 47 | F | Adenocarcinoma | Perineum | Ulcers | Surgery and chemoradiation | Single course | NR | Dehal 2016 |
| Aravind et al., 2013 | 61 | F | Adenocarcinoma | Scalp (recurrent) | NR | Surgery | None | Good | Ahmed 2020 |
| Balta AZ et al., 2013 | 84 | F | Adenocarcinoma | Occiput | Nodule | Chemoradiation | Single course | NR | Hakami 2020 |
| Hashimi et al., 2013 | 70 | M | Adenocarcinoma | Right cheek | NR | Surgery | None | Fair (months NR) | Ahmed 2020 |
| Ozgen et al., 2013 | 65 | M | Adenocarcinoma | Perineum | Nodules | Chemoradiation | Single course | 12 months | Dehal 2016 |
| de Miguel Valencia et al., 2013 | 55 | M | Adenocarcinoma, mucinous | Multiple | Nodules | None | None | NR | Dehal 2016 |
| Kitahara et al., 2014 | 52 | M | Adenocarcinoma, mod. diff. | Perineum | Nodules | Surgery | None | 36 months | Hakami 2020 |
| Kitahara et al., 2014 | 38 | M | Adenocarcinoma, mod. diff. | Perineum | Nodules | Surgery | None | 60 months | Hakami 2020 |
| Kitahara et al., 2014 | 50 | F | Adenocarcinoma, poorly diff. | Perineum | Nodules | Surgery | None | 24 months | Hakami 2020 |
| Sheets et al., 2014 | 78 | M | Adenocarcinoma | Left scapula | NR | Surgery | None | Good (months NR) | Ahmed 2020 |
| Abt et al., 2015 | 67 | M | Adenocarcinoma | Pelvis & scrotum | NR | None | None | Poor (months NR) | Ahmed 2020 |
| Dehal et al., 2015 | 47 | M | Adenocarcinoma, mucinous | Perineum | Nodules | Radiotherapy | Single course | 12 months | Dehal 2016 |
| Fragulidis et al., 2015 | NR | NR | Adenocarcinoma | Scalp | NR | Chemotherapy | None | NR | Ahmed 2020 |
| Ocampo-Candiani et al., 2015 | NR | NR | Adenocarcinoma | Right leg | NR | Chemotherapy | None | NR | Ahmed 2020 |
| Reusser et al., 2015 | 58 | M | Adenocarcinoma | Right flank | Zosteriform | None | None | Poor (months NR) | Ahmed 2020 |
| Yazilitas et al., 2015 | 50 | F | Adenocarcinoma | Forehead | Nodule | None | None | 24 months | Hakami 2020 |
| Goes et al., 2016 | 76 | F | Adenocarcinoma | Right parietal scalp | NR | Surgery and chemotherapy | None | Good | Ahmed 2020 |
| Ha et al., 2016 | 78 | M | Adenocarcinoma | Right parietal scalp | NR | Chemotherapy | None | Poor | Ahmed 2020 |
| Liasis et al., 2016 | 61 | M | Adenocarcinoma, poorly diff. | Perineum | Ulcer | Surgery | None | 60 months | Hakami 2020 |
| Udkoff et al., 2016 | 56 | M | Adenocarcinoma | Scrotum | NR | Chemotherapy | None | Fair (months NR) | Ahmed 2020 |
| Hamid et al., 2017 | 75 | F | Adenocarcinoma, well diff. | Perianal | Nodules | None | None | NR | Hakami 2020 |
| Shah et al., 2017 | NR | NR | Adenocarcinoma | Scalp | NR | Surgery | None | NR | Ahmed 2020 |
| Wang et al., 2017 | 76 | F | Adenocarcinoma, poorly diff. | Back, gingiva | Nodules | None | None | 3 months | Hakami 2020 |
| Abraham et al., 2018 | NR | NR | Adenocarcinoma | Scalp | NR | Surgery | None | NR | Ahmed 2020 |
| Alotaibi et al., 2018 | NR | NR | Adenocarcinoma | Back, scalp, head | NR | Chemotherapy | None | NR | Ahmed 2020 |
| Amarjothi et al., 2018 | NR | NR | Adenocarcinoma | Back / chest wall | NR | Chemotherapy | None | NR | Ahmed 2020 |
| Kemal et al., 2018 | NR | NR | Adenocarcinoma | Forehead | NR | Radiochemotherapy | Single course | NR | Ahmed 2020 |
| Madabhavi et al., 2018 | NR | NR | Adenocarcinoma | Chest wall | NR | Chemotherapy | None | NR | Ahmed 2020 |
| Yagnik et al., 2018 | 38 | M | Adenocarcinoma | Penis & pubis | Nodule + ulcer | None | None | 2 months | Hakami 2020 |
| Hakami et al., 2019 | 45 | M | Adenocarcinoma, mod. diff. | Groin, perineum | Ulcer | Planned RT (died before) | Planned, not delivered | 1 month | Hakami 2020 |
| **Present case (CHUV), 2026** | **36** | **M** | **Adenocarcinoma (germline BRCA1; TP53)** | **Thighs/pubis legs, back contralateral leg (sequential, multi-site)** | **Papules / nodules** | **RT** | **Iterative: 3 courses** | **Alive** |  |

Supplementary table 1: Reported cases of cutaneous metastases from colorectal cancer
